# Excessive Gestational Weight Gain and Pregnancy Outcomes

**DOI:** 10.3390/jcm12093211

**Published:** 2023-04-29

**Authors:** Ksawery Goławski, Wojciech Giermaziak, Michał Ciebiera, Cezary Wojtyła

**Affiliations:** 1 1st Department of Obstetrics and Gynecology, Medical University of Warsaw, 02-015 Warsaw, Poland; 2 The Stanisław Konopka Main Medical Library, 00-791 Warsaw, Poland; 3 Second Department of Obstetrics and Gynecology, Centre of Postgraduate Medical Education, 00-189 Warsaw, Poland; 4 Women’s Health Research Institute, Calisia University, 62-800 Kalisz, Poland

**Keywords:** excessive weight gain, pregnancy, obesity, overweight, pregnancy outcomes

## Abstract

Adequate weight gain during pregnancy is one of the factors for its proper course. Excessive weight gain during this period of a woman’s life is associated with adverse pregnancy outcomes. In this study, we determine the impact of excessive gestational weight gain on pregnancy outcomes. The study is based on the results of a Polish national survey performed between 2011 and 2017 on a group of 10,319 women and 6930 children. Excessive weight gain during pregnancy was associated with higher birthweight and higher prevalence and risk of birthweight over 4500 g (OR 6.92; 95% CI 3.10–15.42), cesarean section/assisted delivery (OR 2.71; 95% CI 1.63–4.49), pregnancy induced hypertension (OR 5.85; 95% CI 3.24–10.57), hospitalization during pregnancy (OR 1.85; 95% CI 1.12–3.04), and the Apgar score in the first minute of neonate’s life in the range of 0–7 (OR 2.65; 95% CI 1.36–5.2). We did not observe the significant difference in premature rupture of membranes and labor inductions. Our study indicates that excessive gestational weight gain is associated with higher risk for adverse pregnancy outcomes.

## 1. Introduction

Appropriate weight is one of the key elements of health. Obesity and overweight are considered a principal public health concern and ranked as the fifth foremost reason for death globally [1]. Unfortunately, this global problem has risen over the last decades. The Global Burden of Disease Study reports an increased prevalence of obesity and overweight in developed and developing countries over the past 30 years [2]. The results of such a state of health is well known. Obesity causes cardiovascular, neurodegenerative, respiratory and autoimmune diseases, hypertension, cancer, diabetes, and many more [1]. Taking the matter of obesity and overweight into consideration, pregnancy is not an extraordinary state.

Both the woman’s weight before pregnancy and its corresponding gain during pregnancy are independent, modifiable risk factors of adverse pregnancy outcomes [3,4,5,6,7]. For this reason, women who are planning pregnancy should try to achieve a healthy weight. Regardless the pre-pregnancy weight, weight gain during pregnancy determines its proper course, and inadequate increases the risk of adverse pregnancy outcomes. These outcomes are related with complications during pregnancy, increased risk of complications during delivery and risk for a baby.

Excessive gestational weight gain (EGWG) is associated with poor pregnancy outcomes, such as higher incidence of cesarean sections, hypertensive disorders during pregnancy or gestational diabetes [8,9,10]. Regarding neonatal consequences, there are examples of influence of EGWG on more frequent admission to neonatal intensive care units or higher incidence of macrosomic neonates [11,12,13,14,15]. Moreover, EGWG has an impact not only on gestational and early postpartum period but is also associated with long-term consequences for both mother and a child, such as childhood or maternal obesity [16,17].

While the optimal body weight of a woman trying to conceive should correspond to the recommended body mass index (BMI) according to World Health Organization (WHO) standards, the adequate weight gain during pregnancy is determined by the standards of the Institute of Medicine (IOM) [18].

In Poland, adequate weight gain during pregnancy is defined in the recommendations of the Polish Society of Gynecologists and Obstetricians regarding the care of pregnant women with diabetes mellitus [19]. These recommendations reflect the abovementioned recommendations of the Institute of Medicine.

The aim of this study is to analyze the impact of excessive gestational weight gain on pregnancy outcomes among population of Polish women.

## 2. Materials and Methods

Analyses of the population of pregnant women in Poland were carried out between 2011 and 2017 within the framework of the Polish Pregnancy-related Assessment Monitoring System organized and carried out by the Chief Sanitary Inspectorate in Poland. This population-based study was carried out in all hospitals in Poland. A group of Polish women and their newborns were investigated during postpartum hospitalization (first days after delivery). The Ethics Committee, a body within the Institute of Rural Health in Lublin, approved the study (reference number 03/2011). All women in Poland who stayed in these hospitals, whose director gave permission to carry out the survey, were deemed eligible for the study. Informed consent was obtained from all women. Participation was voluntary. Thus, the study participants were those women who voluntarily agreed to fill in the survey during designated days of the study. The examination was carried out once in each hospital. The study was conducted simultaneously throughout the whole country, using the structures of the local Sanitary and Epidemiological Stations, as units subordinate to the Chief Sanitary Inspectorate. These types of Stations are located in every poviat in Poland, which allowed for efficient conduction of research throughout the country within one month of the year. In 2011, the study was conducted on one day in each hospital, during the third week of November and in 2012, during the third week of March. In 2017, the study was conducted between the 2 of February and 22 of March. Between 2010 and 2017, 12,076 women took part in Pol-Prams study. The methodology of the current study was the same as in our previous papers [20,21,22,23].

The weight of women before the pregnancy and before labor used for the analyzes was the weight entered to the questionnaire by women themselves. During the instruction on filling in the form by trained interviewers, it was recommended to use the pregnancy card to complete these data. In addition, the medical staff completing part of the questionnaire was instructed to verify this type of data with the pregnancy card. The weight before pregnancy is the weight of woman at the first gynecological visit (up to 10 week of pregnancy). The weight before labor is the weight of woman at the last gynecological visit. These two values were used to calculate gestational weight gain.

In this study we analyzed only term pregnancies, i.e., pregnancies which ended between the full 37 and the incomplete 42 week of gestation. Thus, our sample consisted of 10,319 women and data from 6930 children. In this paper, we used BMI classification according to IOM norms [18] and at the same time, are guided by the Polish recommendations regarding adequate weight gain during pregnancy [19].

### Statistical Analysis

Data were analyzed using IBM SPSS Statistics version 25.0 (IBM Corp., Armonk, NY, USA). The collected data, depending on variable type, were organized using descriptive analysis tables including sample size and percent or mean, median, standard deviation and minimum and maximum values. The Chi square test was used to analyze the dependency between categorical variables. The *p*-value of <0.05 for a two-tailed test was considered as statistically significant. In order to evaluate the effect of weight gain during pregnancy on birthweight, the Mann–Whitney test was implemented. Odds ratios (OR) with confidence intervals (CI) were calculated to analyze the risk for pregnancy outcomes according to excessive weight gain during pregnancy. The adequate weight gain during pregnancy according to IOM norms and Polish recommendations regarding adequate weight gain during pregnancy, were the reference values. The *p*-value of <0.05 for a two-tailed test was considered as statistically significant.

## 3. Results

The mean age of the women was 29 years, and their height was 165.9 cm (Table 1). The mean weight of women before pregnancy was almost 63 kg, while the mean weight of women before delivery was 77.5 kg. The mean time of first gynecological visit in pregnancy was almost 7 weeks of pregnancy.

Table 2 presents women’s BMI distribution according to WHO and IOM categories. Over 68% of women had healthy weight range before pregnancy according to WHO norms. The second largest group had overweight (16.8%). Underweight was found in 9% of women and obesity in 5.5%. Almost 99% of women had adequate weight gain during pregnancy. At the same time, 1.2% of women were characterized by excessive weight gain during pregnancy (Table 3). Almost 93% of women whose weight gain during pregnancy was excessive, were the women diagnosed with overweight or obesity before pregnancy. However, taking into account the BMI groups according to the IOM, it was found that slightly more than 62% of all analyzed women had a BMI before pregnancy in the healthy range. In the group of women who were underweight, overweight and obese, there were 21.5%, 8.6%, and 7.7% of all analyzed women, respectively.

The mean birthweight of children, whose mothers had adequate weight gain during pregnancy was 3440.6 g. The mean birthweight of children, whose mothers gained weight over the recommended norms was 3687.3 g. The influence of the woman’s body weight gain during pregnancy on the birthweight of the child is presented in Table 4.

Table 5 shows the effect of excessive gestational weight gain during pregnancy on other perinatal outcomes. The majority of women with EGWG had the caesarean section. There were no cases of assisted deliveries (using forceps or vacuum extractor). There were no significant differences in the rates of premature rupture of membranes (PROM) and labor inductions. However, the prevalence of pregnancy-induced hypertension (PIH) increased significantly. Among women with adequate weight gain during pregnancy, the percentage of cases with this complication was 3.5%, while in the group of women with EGWG, it increased to 17.5%. The percentage of women hospitalized during pregnancy also increased from 37.3% (adequate weight gain) to 52.4% (EGWG). We also observed significant differences between the groups in Apgar scores at first minute of neonate’s life. Among women with adequate weight gain during pregnancy, the percentage of children with an Apgar score of 7 or less, at first minute of life, was found in 5.2%. Among women with EGWG, this percentage was 12.7%.

Table 6 shows the risk of selected complications in the group of women with EGWG compared to the group of women with adequate weight gain during pregnancy. There was no change in the risk of low birthweight (less than 2500 g), PROM, labor induction, diabetes or an Apgar score under 8 in 10th minute of neonate’s life. However, we observed significantly higher risk of birthweight over 4500 g (OR 6.92; 95% CI 3.10–15.42), cesarean section/assisted delivery (OR 2.71; 95% CI 1.63–4.49), PIH (OR 5.85; 95% CI 3.24–10.57), hospitalization during pregnancy (OR 1.85; 95% CI 1.12–3.04), and the Apgar score in the 1st minute of neonate’s life in the range of 0–7 (OR 2.65; 95% CI 1.36–5.2).

## 4. Discussion

In our study, we focused on influence of excessive gestational weight gain on pregnancy outcomes. Characteristics of our study group seem to correspond with Western European study concerning EGWG made by Gaillard et al. based on Dutch population [24]. Maternal mean age is around 30, height around 166 cm, structure of maternal BMI (based on WHO guidelines) before pregnancy is also similar. On the other hand, the latter issue is substantially different in American study [25], enrolling over 8500 women, where obesity (BMI ≥ 30 kg/m^2^) accounted for 22% of study group in comparison to 8.8% of Dutch population [24] and 5.5% in our study, which can possibly be explained by differences in obesity prevalence among Europeans and Americans [26,27].

Our results are in line with Dude et al. [25] and Wu et al. [28], where cesarean section incidence and its odds ratio were significantly higher among patients with excessive weight gain. PIH incidence in our population was higher among women with excessive weight gain, whereas Dude et al. presented higher incidence of general hypertensive disorders, including not only PIH, but also preeclampsia and HELLP syndrome [25]. The Avon longitudinal study, which prospectively evaluated a cohort of 12,522 women found that EGWG was associated with increased risks of gestational hypertension and preeclampsia compared with weight gain within the recommended range [29]. Gaillard et al. enrolling over 6000 women, showed that EGWG is associated with a higher risk of gestational hypertension what corresponds with our results [24]. It is worth mentioning that odds ratios for cesarean section and PIH from our study are substantially higher (2.71, 95% CI: 1.63–4.49, and 5.85, 95% CI: 3.24–10.57, respectively) in comparison with studies mentioned above (1.56, 95% CI: 1.39–1.76, and 2.07, 95% CI: 1.43–2.99, respectively). We did not observe a higher prevalence and odds ratio of labor induction or PROM, but the course of pregnancy in our study was associated with higher risk of hospitalization in pregnancy, which may be correlated with higher risk of PIH.

Regarding neonatal outcomes, we found statistically significant difference for Apgar score in the first minute of life, what can be correlated with later neonatal intensive care unit admission, for which, Dude et al. showed similar statistical significance in terms of EGWG [25]. Furthermore, women with excessive gestational weight gain showed significant increase in the risk of birthweight exceeding 4500 g, being in line with several other studies [11,12,13,14,15].

This paper is one of a series of publications analyzing the results of the Pol-Prams study. This series aims to analyze health behaviors of pregnant women in Poland, and to analyze the impact of these behaviors and environmental factors on pregnancy outcomes. In one of the first publications, we also described in detail the research methodology [23]. The results obtained so far indicate that the vast majority of Polish women limit their physical activity during pregnancy. Therefore, pregnant women mostly lead a sedentary lifestyle or engage in low-intensity physical activity [30]. We observed a positive effect of total physical activity of women during pregnancy on birthweight. We observed the greatest differences in this respect in the case of moderate-intensity activity. Thus, the higher the women’s energy expenditure, the higher birthweight of the child was observed, but it was still within the normal range. We also observed higher percentage of vaginal birth in the group of women with higher energy expenditure during pregnancy. In addition, a lower incidence of low-birthweight and premature births was observed in the group of women whose energy expenditure during pregnancy was higher [31]. Almost 20% of woman in Poland smoked cigarettes before pregnancy. The percentage of women actively smoking during pregnancy was much lower and amounted to less than 7%. Nevertheless, exposure to passive smoking was declared by 40% of pregnant women. Regardless of the period of smoking (before pregnancy, in the first three months of pregnancy or in the last three months of pregnancy), it was associated with a decrease in birthweight compared to women who did not smoke cigarettes, and the strength of this effect depended on the dose of smoked cigarettes per day. Smoking cigarettes in the period preceding pregnancy and quitting smoking at the time of obtaining information about pregnancy did not eliminate this negative effect. In addition, active smoking during pregnancy was associated with an increased risk of low birthweight. Passive smoking during pregnancy had an equally negative impact on the birthweight of the child. However, there was no significant increase in the risk of low birthweight [32]. In a subsequent study, we assessed the impact of air pollution on pregnancy outcomes. We observed that the birthweight in a group of women living in an environment with air pollution exceeding the permissible average standard of annual PM2.5 concentration in Europe was decreased. In this group of women, we also observed higher percentage of low birthweight. There was also higher risk of one of the following pathologies: low birthweight, PROM, Apgar score <8 and fetal abnormalities [21]. In another paper, the impact of obesity on the course of pregnancy and pregnancy outcomes was assessed, and the impact of changes in the perinatal care system in Poland on the course and outcomes of pregnancy. For this purpose, two groups of women who gave birth over a period of 5 years were compared. This was dictated by a number of changes in perinatal care of pregnant women that appeared in Poland between 2012 and 2017. The percentage of obese women increased over the analyzed 5 years and in 2017, it was 7.5%. Overweight and obesity negatively affected the course of pregnancy and pregnancy outcomes. A positive effect of changes in perinatal care on the course of pregnancy was observed. On the one hand, the percentage of women diagnosed with diabetes increased, which resulted from the improvement in diagnostics. On the other hand, an improvement in the control of body weight of women and the baby was observed [33].

In our group, the vast majority of women with EGWG were obese or overweight before pregnancy. Both overweight and obesity are risk factors for the development of pathologies during pregnancy and during labor. Taking this into account, there was a possibility of bias, which could be a consequence of excessive BMI before pregnancy and not EGWG. However, in our additional analyses performed only in a group of women with overweight and obesity before pregnancy, we observed almost the same impact of EGWG on pregnancy outcomes. The only difference was that association between EGWG and hospitalization in pregnancy was not statistically significant (*p* = 0.08), but the pattern was the same. In our previous study, we analyzed an impact of obesity on pregnancy outcomes [33]. We found an increased prevalence of cesarean section in a group of obese women, but this observation was not statistically significant. Obesity was connected with higher birthweight and the risk of diabetes. There was no relation between obesity and labor induction and birth defects. The current paper therefore complements our previous analyses. We observed similar relationships, but the difference was that the percentage of women with EGWG had a cesarean section much more often than the total group of obese women in our previous paper and this relation was statistically significant. In addition, the increase in birthweight among women with EGWG was greater than in the case of obesity alone.

Our study has several limitations. We are aware that weight gain during pregnancy is a complex issue. It does not only influence pregnancy outcomes and general health condition but is under influence itself. Physiological factors seem to have an obvious impact, but the psychological, environmental, behavioral, family and cultural impacts can also influence gestational weight gain (GWG) [18]. However, after inclusion of over 10,000 women into the study from all around the 40-million country, we hope that the extent of abovementioned factors influence could be equalized. Another limitation is possible recall bias. Our results come from women’s declarations about their weight. They were not weighted by medical professionals at the end of pregnancy, which would possibly standardize the values. Nevertheless, before completing the form, the women were instructed by trained interviewers to use the pregnancy card to complete these data. In addition, the medical staff was asked to verify this type of data. Assessment of the weight of a pregnant woman is an obligatory point of every visit with a gynecologist during pregnancy. This data is entering to individual pregnancy card. Considering the above, there is a small probability of obtaining incorrect data.

Another limitation is the inability to include other pregnancy pathologies, such as intrauterine growth restriction or preeclampsia, in the analyses. The reason is the lack of information about it in our survey. Moreover, we focused on excessive gestational weight gain during whole pregnancy, when Gaillard et al. and Wu et al. distinguished gestational weight gain for early and late pregnancy [24,28].

Not only the EGWG has negative effect on pregnancy outcomes. In general, inadequate weight gain, which consists also insufficient weight gain, can lead to complications [10]. Dude et al. analyzed a group of 2945 mothers with low GWG and showed that they had higher odds of having a baby with low birthweight (adjusted odds ratio for small for gestational age was 1.64, 95% CI: 1.37–1.96). On the other hand, authors observed that this group of women had lower odds of hypertensive disorders, cesarean delivery, and a large for gestational age (LGA) [11]. Wu et al. in his prospective cohort study showed that lower GWG was not significantly associated with adverse pregnancy outcomes, compared with the average GWG [28].

In 2009, the Institute of Medicine updated its recommendations for adequate weight gain during pregnancy [18]. It was the result of the analysis of new scientific data on the impact of inadequate GWG on pregnancy outcomes and the growing obesity epidemic. The main change was the narrowing of the optimal weight gain during pregnancy for women who were overweight and obese in the period preceding pregnancy. The abovementioned document states that the recommendations are addressed to the American population. The Polish Society of Gynecologists and Obstetricians, following the recommendations of the IOM, determined the optimal weight gain during pregnancy for the population of pregnant women in Poland [19]. It reflects the American recommendations with the difference that for obese women the recommended weight gain during pregnancy is less than 7 kg, not 5–9 kg as in the American population. Body Mass Index Classification was also adopted from IOM norms.

Undoubtedly, we live in an obesity epidemic that does not spare women in their reproductive years [18]. Epidemiological data from the USA indicate that in this population the percentage of women with obesity class III (BMI > 40 kg/m^2^) exceeds the percentage of women with underweight [18]. According to WHO estimates, 39% of adults in the world are overweight and 13% are obese. The number of obese people in the world has tripled since 1975 [34]. In Poland, there is also an upward trend in obesity [35,36]. Currently, approximately 46% of adult women are overweight or obese [36]. The above data raise concerns in the context of the results of our study, which indicates that the group of women among whom the highest percentage of excessive weight gain during pregnancy was observed were women with overweight and obesity in the pre-pregnancy period. Together they accounted for approximately 93% of all observed cases of excessive weight gain during pregnancy. Unfavorable trends indicating a gradual increase in the percentage of obese women in Poland suggest that this problem will intensify in the coming years. This should raise concerns in the context of data on the prevalence of other anti-health behaviors of pregnant women, which have a negative impact on pregnancy outcomes. These include smoking [32], alcohol consumption [37], and insufficient physical activity during pregnancy [30,31]. Environmental factors, such as air pollution, have an equally significant impact [21]. There is a need to intensify activities aimed at improving public awareness of unhealthy behaviors during pregnancy, and minimizing the impact of environmental factors. All of them have an impact on the health of future generations.

## 5. Conclusions

Based on the study results, we observed higher incidence and higher risk of several adverse pregnancy outcomes in women with excessive weight gain during pregnancy. EGWG was related with the risk of macrosomia (birthweight of over 4500 g), cesarean section, pregnancy induced hypertension, hospitalization in pregnancy, lower Apgar score, and higher birthweight. Excessive gestational weight gain should be treated as another unhealthy behavior in pregnancy, next to smoking, alcohol consumption or lack of physical activity. Therefore, every obstetrician-gynecologist in her/his daily practice should encourage pregnant women to maintain adequate weight gain in pregnancy.

## Figures and Tables

**Table 1:** Characteristics of the study population.

Variable	N	Mean	SD	Median	Minimum	Maximum
Age	10,319	29.0	5.18	29.0	14.5	51.1
Height (cm)	10,093	165.9	5.89	165.0	139.0	198.0
First gynecological visit (weeks of pregnancy)	4309	6.6	3.25	6.0	1.0	40.0
Weight before pregnancy	7259	62.7	11.62	60.0	34.9	162.0
Weight before delivery	7259	77.5	12.75	75.5	43.5	180.0

N, number; SD, standard deviation.

**Table 2:** Women’s BMI distribution according to WHO and IOM categories.

Characteristics	Weight Gain During Pregnancy						*p*-Value
	Total		Adequate		Excessive		
	N	%	N	%	N	%	
**BMI before pregnancy** (WHO norms)							<0.05
underweight (<18.5 kg/m^2^)	647	9.0%	646	9.1%	1	1.2%	
healthy weight range (18.5–24.9 kg/m^2^)	4922	68.6%	4917	69.4%	5	6.0%	
Overweight (25.0–29.9 kg/m^2^)	1207	16.8%	1178	16.6%	29	34.5%	
Obesity (≥30 kg/m^2^)	394	5.5%	345	4.9%	49	58.3%	
**BMI before pregnancy** (IOM norms)							<0.05
underweight (<19.8 kg/m^2^)	1542	21.5%	1541	21.8%	1	1.2%	
healthy weight range (19.8–26.0 kg/m^2^)	4461	62.2%	4456	62.9%	5	6.0%	
Overweight (26.1–29.0 kg/m^2^)	614	8.6%	612	8.6%	2	2.4%	
Obesity (>29 kg/m^2^)	550	7.7%	474	6.7%	76	90.5%	

BMI, body mass index; WHO, World Health Organization; IOM, Institute of Medicine.

**Table 3:** Weight gain during pregnancy.

Weight Gain	N	%
Adequate	7086	98.8%
Excessive	84	1.2%
Total	7170	100.0%

N, number.

**Table 4:** Birthweight (grams) according to mothers weight gain during pregnancy.

Weight Gain	N	Mean	SD	Median	Minimum	Maximum	*p*
**Adequate** **Excessive**	6847	3440.6	483.90	3450.0	380.0	5660.0	<0.05
	83	3687.3	600.66	3670.0	2115.0	5220.0	

N, number; SD, standard deviation.

**Table 5:** Selected perinatal outcomes according to weight gain during pregnancy.

Variable	Weight Gain during Pregnancy				*p*-Value
	Adequate		Excessive		
	N	%	N	%	
**Type of labor**					<0.05
Vaginal	2971	63.5%	25	39.1%	
Cesarean section	1657	35.4%	39	60.9%	
Assisted (forceps, vacuum)	54	1.2%	0	0.0%	
**PROM**					0.694
No	3062	65.6%	41	63.1%	
Yes	1605	34.4%	24	36.9%	
**Labor induction**					0.415
No	3311	70.4%	49	75.4%	
Yes	1391	29.6%	16	24.6%	
**PIH**					<0.05
No	6593	96.5%	66	82.5%	
Yes	239	3.5%	14	17.5%	
**Hospitalization in pregnancy**					<0.05
No	2892	62.7%	30	47.6%	
Yes	1722	37.3%	33	52.4%	
**Apgar score in 1st min.**					<0.05
8–9	6227	94.8%	69	87.3%	
0–7	340	5.2%	10	12.7%	
**Apgar score in 10th min.**					0.158
8–9	4622	98.8%	57	96.6%	
0–7	55	1.2%	2	3.4%	

PROM, premature rupture of membranes; PIH, pregnancy induced hypertension.

**Table 6:** The risk of selected perinatal outcomes in women with excessive weight gain during pregnancy (a group of women with adequate weight gain during pregnancy was a reference category for each of the variable).

Variables	Odds Ratio	95% Confidence Interval		*p*-Value
		Lower	Upper	
Low birthweight (<2500 g)	0.93	0.23	3.79	0.914
Birthweight > 4500 g	6.92	3.10	15.42	<0.05
Cesarean section or assisted delivery	2.71	1.63	4.49	<0.05
PROM	1.12	0.67	1.85	0.670
Induced labor	0.78	0.44	1.37	0.384
PIH	5.85	3.24	10.57	<0.05
Diabetes	1.69	0.68	4.22	0.258
Hospitalization in pregnancy	1.85	1.12	3.04	<0.05
Apgar score in 1st min.	2.65	1.36	5.20	<0.05
Apgar score in 10th min.	2.95	0.70	12.38	0.140

PROM, premature rupture of membranes; PIH, pregnancy induced hypertension.

## Data Availability

Not applicable.
